# Transcriptomic data of mature oil palm basal trunk tissue infected with *Ganoderma boninense*

**DOI:** 10.1016/j.dib.2019.104288

**Published:** 2019-07-19

**Authors:** Nur Qistina Othman, Suhaila Sulaiman, Yang Ping Lee, Joon Sheong Tan

**Affiliations:** FGV R&D Sdn. Bhd., FGV Innovation Centre (Biotechnology), PT 23417 Lengkuk Teknologi, 71760 Bandar Enstek, Negeri Sembilan, Malaysia

**Keywords:** Basal stem rot, *Ganoderma boninense*, Oil palm, Infection

## Abstract

To date, *Ganoderma boninense* is known to be the causal agent of basal stem rot (BSR) disease in oil palm (*Elaeis guineensis*). This disease causes rotting in the roots, basal and upper stem of oil palm. Infection causes progressive destruction of the basal tissues at the oil palm trunk and internal dry rotting, particularly at the intersection between the bole and trunk. Molecular responses of oil palm during infection are not well study although this information is crucial to strategize effective measures to control or eliminate BSR. Here we report three sets of transcriptome data from samples of near-rot section of basal stem tissue of oil palm tree infected with *G. boninense* (IPIT), healthy section of basal stem tissue of the same *G. boninense* infected palm (IPHT) and the healthy section of basal stem tissue of the healthy palm (HPHT). The raw reads were deposited into NCBI database and can be accessed via BioProject accession number PRJNA530030.

**Table:** Specifications Table

Subject area	Biology
More specific subject area	Molecular biology
Type of data	Transcriptome data
How data was acquired	Paired-end reads of *G. boninense* transcriptome were sequenced using Illumina HiSeq 2000
Data format	Raw sequence in FASTQ format
Experimental factors	Total RNA was extracted from a pair of healthy and infected clonal mature palm
Experimental features	Total RNA of infected and non-infected matured oil palm basal stem were extracted using method by Saidi *et al*., 2009 [1]. The extracted RNAs were used for transcriptome sequencing using Illumina HiSeq 2000 platform, (Illumina, USA).
Data source location	Serting Hilir, Negeri Sembilan, Malaysia
Data accessibility	Raw reads in FASTQ format were deposited in NCBI SRA database with accession number SRX5608988, SRX5608989 and SRX5608990 (https://www.ncbi.nlm.nih.gov/sra/?term=SRX5608988, https://www.ncbi.nlm.nih.gov/sra/?term=SRX5608989, https://www.ncbi.nlm.nih.gov/sra/?term=SRX5608990)

**Table:** 

**Value of the data** • The data reported here is beneficial to elucidate the plant-pathogen interaction for matured oil palm tree that is naturally infected with *G. boninense* in an open oil palm estate. • This data information is crucial to strategized effective measures against BSR in the future. • The data allows further comparative analysis to identify candidate genes of interest that could play significant roles in oil palm defense system. • With the accessible data, more subsequent projects can be accelerated to elucidate infection pathway of *G. boninense* in oil palm as well as identification of genes, proteins or metabolites involved during the infection process that may facilitate the development of possible solutions against *G. boninense.*

## 1 Data

Three sets of transcriptome data generated from the cDNA libraries were prepared from the total RNA extracted from three different samples as mentioned above. The data sets were named as IPHT (the healthy section of infected oil palm), IPIT (the near-rot section of infected oil palm) and HPHT (the cross section of healthy tissue section of healthy oil palm tree).

## 2 Experimental design, materials and methods

### 2.1 Sampling materials

A pair of healthy and infected clonal mature palm was selected from Serting Hilir field research station, Negeri Sembilan, Malaysia owned by Felda Agricultural Services Sdn Bhd. Briefly, the bark was marked according to the health status. The healthy section (IPHT) and near-rot section (IPIT) of infected oil palm were collected from the cross section of the basal stem. The cross section of healthy tissue section of healthy oil palm tree (HPHT) was also collected. The healthy palm was determined by healthy oil palm phenotype with no *G. boninense* fruiting body attached at the basal stem and the palm does not exhibit stress-related symptoms phenotypically. All collected basal stem tissues were immediately submerged in liquid nitrogen and kept at −80 °C freezer until further processing for RNA extraction.

### 2.2 RNA extraction and quality assessment of total RNA

Total RNA of matured basal stem samples; IPHT, IPIT and HPHT were extracted using method from Saidi *et al*., 2009 [1]. The quality of the extracted RNAs was determined using Bioanalyzer (Agilent Technologies, USA) and UV absorbance readings (NanoDrop, Thermo Fisher Scientific Inc., USA). The extracted RNAs were used for Illumina's HiSeq 2000 transcriptome sequencing.

### 2.3 Transcriptome sequencing

Messenger RNA isolation and cDNA synthesis were performed using TruSeq RNA Sample Preparation Kit (Illumina, USA) and SuperScript II Reverse Transcriptase (Invitrogen, USA) by following the manufacturers' protocol, respectively. The amount of synthesised cDNA was measured using Qubit 2.0 DNA Broad Range Assay (Invitrogen, USA). A minimum of 15 ng cDNA was fragmented using Covaris S220 (Covaris Inc, USA) to a targeted size of 200–300bp. The fragmented cDNA was then end repaired, ligated to Illumina TruSeq adapters, and PCR-enriched using TruSeq RNA Sample Preparation Kit (Illumina, USA) by following the manufacturer's protocol. The final sequencing libraries were quantified using KAPA kit (KAPA Biosystem, USA) on Agilent Stratagene Mx-3005p quantitative PCR (Agilent Technologies, USA) and sizes were confirmed using Agilent BioAnalyzer High Sensitivity DNA Chip (Agilent Technologies, USA). The resulting libraries were sequenced using an Illumina flow cell and 209 cycles on the Illumina HiSeq 2000 platform (Illumina, USA).

### 2.4 Pre-processing of RNA-seq raw data and transcriptome mapping

**Fig. 1:**
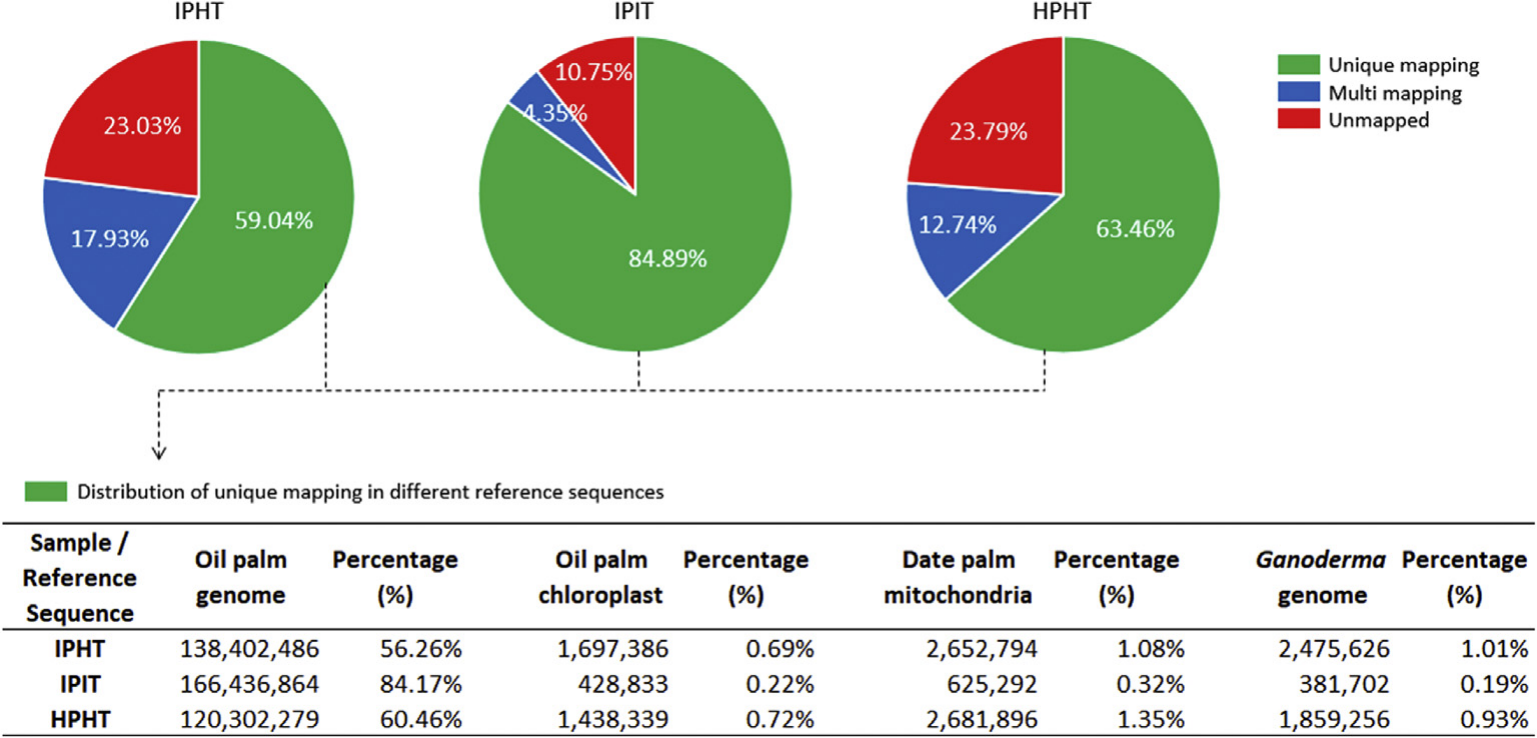
Statistics of raw data mapping to different reference sequences (oil palm genome, oil palm chloroplast, date palm mitochondria and *Ganoderma* genome) for sample IPHT, IPIT and HPHT.

The quality of RNA-seq raw data was assessed using FastQC [2] and pre-processed with FASTX-Toolkit [3] to trim off adaptor sequences and low quality bases (<Q20). Reads containing unknown bases or reads that were shorter than 30 bp after trimming were discarded to obtain clean reads (Table 1). The high quality reads of each sample were aligned to reference sequences consisting of *E. guineensis* genome [4], oil palm chloroplast genome [5], date palm mitochondrial genome [6] as well as *Ganoderma boninense* genome [7] (Fig. 1). The read alignment was carried out using TopHat v2.0.12 [8] and Bowtie v2.2.1 [9] as algorithmic core [params: -segment-length 45 --read-mismatches 2].

**Table 1:** Statistics of raw reads from three samples before and after pre-processing.

Sample name	Description	Details	Before pre-processing	After pre-processing		
				Total reads	Paired reads	Orphan reads
IPHT	Infected Palm, Healthy tissue	Total number of read	276,546,920 (100.00%)	246,029,758 (88.96%)	245,994,098 (88.95%)	35,660 (0.01%)
		Total read size	27,654,692,000 (100.00%)	21,610,020,425 (78.14%)	21,606,913,722 (78.13%)	3,106,703 (0.01%)
IPIT	Infected Palm, Intersection zone	Total number of read	214,112,784 (100.00%)	197,773,317 (92.37%)	197,742,548 (92.35%)	30,769 (0.01%)
		Total read size	21,411,278,400 (100.00%)	18,120,714,405 (84.63%)	18,117,911,395 (84.62%)	2,803,010 (0.01%)
HPHT	Healthy Palm, Healthy tissue	Total number of read	224,285,692 (100.00%)	199,014,398 (88.73%)	198,985,062 (88.72%)	29,336 (0.01%)
		Total read size	22,428,569,200 (100.00%)	17,674,879,482 (78.81%)	17,672,289,541 (78.79%)	2,589,941 (0.01%)
